# 113. Improving Transitions-of-Care for Patients Discharged on High-Risk Antimicrobial Therapy

**DOI:** 10.1093/ofid/ofab466.315

**Published:** 2021-12-04

**Authors:** Ryan Zabrosky, Ellen C Rubin, Erica Liu, Karrine Brade, Hope Serafin, Spencer E Sutton

**Affiliations:** Boston Medical Center, Quincy, Massachusetts

## Abstract

**Background:**

Providing effective transitions-of-care (TOC) services improves outcomes for patients discharged on high-risk medications. Literature has shown that successful TOC for certain antimicrobials reduces hospital readmissions, medication errors, and improves post-discharge follow-up and laboratory monitoring. Prior to this quality improvement (QI) initiative, there was no formal TOC process for patients discharged on high-risk antimicrobial therapy (HAT) at our institution. Without standardization, only 55.1% of patients discharged on HAT had successful TOC. The aim of this initiative was to develop and implement a TOC protocol in at least 90% of patients discharged on HAT.

**Methods:**

This QI initiative utilized the Institute of Healthcare Improvement model for improvement. A workgroup of key stakeholders developed a protocol to identify and standardize TOC services provided to patients discharged on HAT. Successful protocol completion was achieved if the following process metrics were evaluated, obtained, and documented prior to discharge: baseline laboratory values, pharmacokinetic monitoring, appropriate intravenous access, drug-drug interactions, medication availability, discharge medication counseling, and formal pharmacist documentation in a discharge note. Outcome metrics included referral to outpatient infectious disease (ID) follow-up, 90-day readmissions, and successful TOC. Balancing metrics included pharmacist time and protocol initiation for patients not discharged on HAT.

**Results:**

Between October 2020 and May 2021, 218 patients met protocol inclusion criteria. Of these, 203/218 (93.1%) were appropriately identified with the new TOC process. The protocol was successfully followed in 78.9% of patients identified. Readmission rates were 42.8%, which was unchanged from baseline. Inpatient ID involvement increased from 80.9% to 95.7% and referral to outpatient ID follow-up from 59% to 94.8%.

**Conclusion:**

This newly developed TOC protocol successfully identifies patients discharged on HAT, improves provision of TOC services to these high-risk patients, and significantly improves the rate of infectious disease involvement while inpatient and after discharge.

**Disclosures:**

**All Authors**: No reported disclosures

